# Genus *Bithynia*: morphological classification to molecular identification

**DOI:** 10.1186/s13071-024-06591-0

**Published:** 2024-11-30

**Authors:** Guoyang Huang, Xiaohong Peng

**Affiliations:** 1 Guangxi University Key Laboratory of Pathogenic Biology, Guilin Medical University, Guilin, Guangxi People’s Republic of China; 2 Guangxi Key Laboratory of Molecular Medicine in Liver Injury and Repair, The Affiliated Hospital of Guilin Medical University, Guilin, Guangxi People’s Republic of China

**Keywords:** Genus *Bithynia*, Morphology and anatomy, Multi-site enzyme electrophoresis, DNA barcoding technology, Metagenomics

## Abstract

Snails of the genus *Bithynia*, whose primary habitat is slow-flowing ponds and ditches, serve as the first intermediate hosts of liver fluke. Currently, approximately 200 million individuals worldwide are at risk of liver fluke infection, yet questions still persist regarding the taxonomic identification of *Bithynia* genus, a crucial player in the transmission of this disease. Accurate taxonomic classification of the *Bithynia* genus could significantly enhance current understanding of the disease’s transmission mechanisms. In this article we comprehensively review the extensive research conducted on *Bithynia* genus, spanning past inquiries up to the latest findings. The primary emphasis is placed on exploring the taxonomic identification of this genus within various technological settings. We then present a consolidated analysis of the morphological taxonomic identification methods, highlighting their strengths and limitations. We also introduce a novel perspective on the future direction of identification and classification efforts for the members of this genus, emphasizing the crucial role *Bithynia* plays in the epidemiological cycle of liver fluke transmission. We conclude by urging researchers to prioritize the significance of the members of this genus in the epidemiological cycle of liver fluke transmission and in control measures for disease dissemination, within the context of the vector organisms.

**Graphical Abstract:**

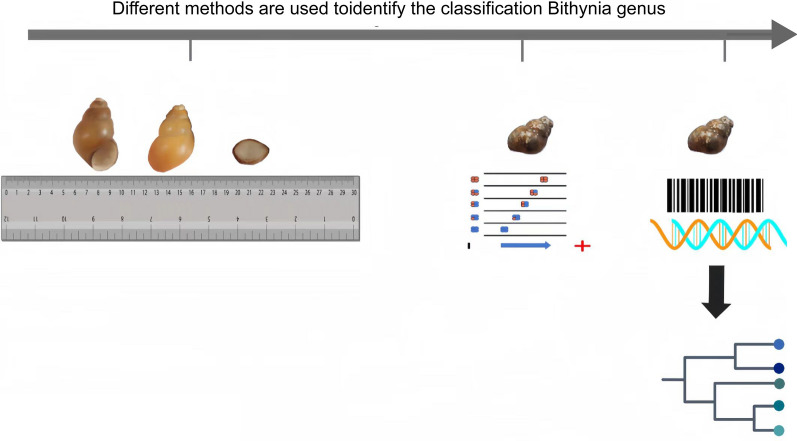

## Background

The genus *Bithynia* (Mollusca: Gastropoda: Mesogastropoda: Bithyniidea), can be taxonomically classified into two subgenera, namely *Digoniostoma* and *Gabbia* [1, 2]. This genus is widely distributed in Europe, Asia, Africa and other regions, and is primarily found in habitats such as slow-moving or stationary rivers, ditches, lakes, farmlands and reservoirs [3, 4]. While the specific impacts of the genus *Bithynia* on the natural environment and other organisms remain incompletely documented, its well-established role as the primary intermediate host of liver fluke diseases underscores its significance in the transmission of this group of diseases [5].

Members of the genus *Bithynia*, including *B. funiculate*, *B. siamensis goniomphalos*, *B. siamensis siamensis*, *B. fuchsiana* and *B. fuchsianus*, have been identified as the first intermediate host of liver flukes in Asia [6]. In Southeast Asia, *Bithynia siamensis siamensi*s, *Bithynia siamensis goniomphalos* and *Bithynia funicaulata* play a crucial role as first intermediate hosts of liver fluke [7]. Similarly, in Siberia, *Bithynia* species, including *B. leachii*, *B. troscheli*, *B. inflata* and *B. tentaculata*, are essential first intermediate hosts [8]. These findings underscore the pivotal role played by snails of genus *Bithynia* in the life-cycle of liver fluke parasites and emphasize the need for further research to elucidate the broader ecological implications.

The main liver fluke species that can infect humans are *Clonorchis sinensis*, *Opisthorchis viverrini* and *Opisthorchis felineus*. *Clonorchis sinensis* is prevalent in several countries in the Far East region, including Korea, parts of Russia, China and Vietnam. *Opisthorchis viverrini* is primarily found in Southeast Asian countries, such as Thailand, while *O. felineus* is endemic to regions of Northern Asia and Eastern Europe [9–11].

**Fig. 1:**
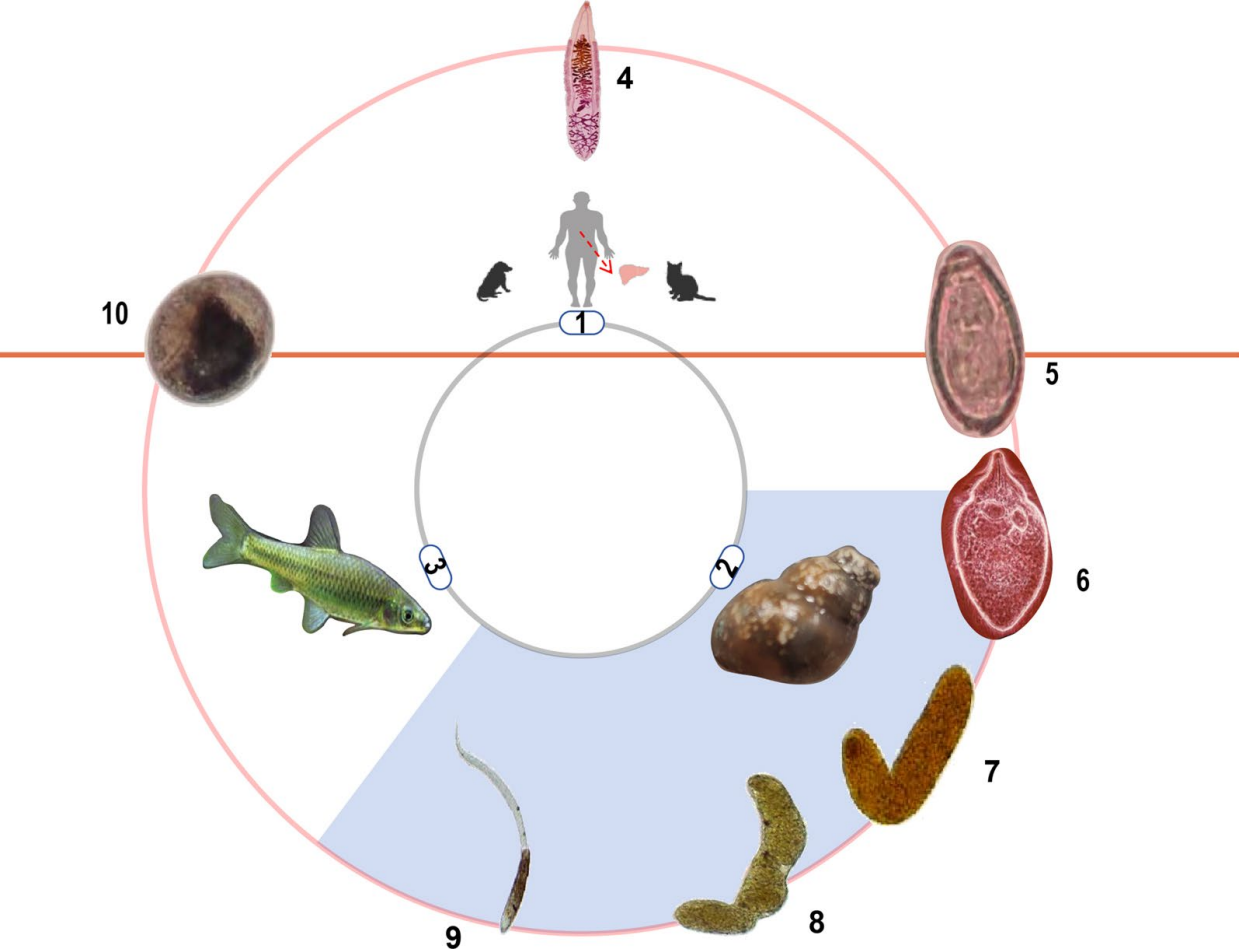
Life-cycle of the human liver fluke *Clonorchis sinensis*. The encircled numbers denote the different intermediate/final hosts (1–3) and the life-cycle stages of the liver fluke (4–10). 1, Final host (human, cat, dog); 2, first intermediate host (*Bithynia* spp.); 3, second intermediate host (family Cyprinidae [freshwater fish/minnow family]); 4, adult worm; 5, egg; 6, miracidium; 7, sporocyst; 8, rediae; 9, cercaria; 10, metacercaria. The life-cycle of *C. sinensis* begins when the metacercariae, residing within the fish, are ingested by the definitive host. In the duodenum of this host, the metacercariae excyst due to the action of digestive juices and develop into adults within the hepatobiliary duct. Once the worms reach maturity, their eggs are excreted into the feces of the definitive host and enter the water, where they are ingested by the first intermediate host, a freshwater snail. Within the snail, they pass through four stages (miracidium, sporocyst, rediae, cercaria) before the cercaria are released from the snail's anus, infecting freshwater fish. Inside the fish, they develop into metacercariae, thus completing the entire life-cycle by infecting the definitive host

The life-cycle of liver flukes necessitates three hosts. Freshwater fish and shrimp infected with metacercariae are ingested by the final mammalian host. Under the influence of digestive fluid, these metacercariae shed their cysts and migrate to the liver and bile ducts to develop into adults. The eggs produced by the adult worms are released into the water with feces and ingested by the first intermediate host, freshwater snails, such as *Alocinma longicornis*, *Parafossarulus striatulus*, *B. funiculate*, *B. siamensis goniomphalos* and *B. fuchsianus*. These eggs develop into cercaria within the snails. Subsequently, the cercaria infect the second intermediate host, which includes freshwater fish and shrimp [12, 13] (Fig. 1).

Minor liver fluke infections are often asymptomatic, while severe infections can lead to symptoms such as malaise, nausea, dyspepsia, abdominal discomfort, diarrhea or abdominal pain, particularly in the right upper abdomen. Previous studies have established a link between liver fluke infection and cholangiocarcinoma, although the precise mechanism underlying carcinogenesis remains incompletely understood and involves complex interactions among various mechanistic pathways [14–18].

In 2009, *C. sinensis* was classified as a class I biocarcinogen [19], indicating its high carcinogenic potential. By 2015, it was estimated that over 200 million individuals worldwide were exposed to the risk of liver fluke infection, with approximately 15 million patients suffering from Clonorchis disease in Asia. Clonorchiasis (also known as Chinese liver fluke disease) is particularly prevalent in several Asian countries, including China, Thailand, South Korea and Vietnam [20–22]. Among the Asian countries, China has been reported to have the highest number of infected individuals, with an estimated 13 million people affected [5]. *Clonorchis sinensis* is the primary endemic species in China, and the vast majority of species within the genus *Bithynia* serve as the first intermediate host for this parasite [23]. Members of the genus *Bithynia* play a crucial role in the life-cycle of *C. sinensis*, acting as a key link in the transmission of the parasite to humans. As such, these snails have become a significant factor in public health efforts to prevent and control the spread of liver fluke disease among humans. Understanding the ecology, immunology and behavior of these snails is essential in the context of developing effective strategies to interrupt the parasite's transmission cycle and ultimately mitigate the burden of Clonorchis disease in endemic regions.

While the role of snails of *Bithynia* genus in *C. sinensis* transmission cannot be overstated, the current taxonomic classification of species within this genus remains a challenge. The lack of clear taxonomic boundaries and international standardization for species identification has led to inconsistencies and confusion in research, affecting the reliability of conclusions. This is particularly problematic in the study of liver fluke infection patterns, as variations between species may be obscured by imprecise classification [24, 25]. The advent of cutting-edge technologies in computer science, molecular biology and artificial intelligence (AI) has heralded a new era in species classification. These advancements have armed researchers with sophisticated tools to amass extensive data on species morphology and genetic makeup, significantly enhancing our comprehension of taxonomic intricacies. This evolution in our understanding of the genus *Bithynia* is documented, emphasizing the shift in approach from morphological physical characteristics to genetic diversity, facilitating a more nuanced classification.

In essence, the objective of this review is to illuminate the critical role of robust taxonomic practices in advancing the public health fight against liver flukes. By refining our classification methods, we not only enhance academic research but also contribute to the development of evidence-based public health policies that can mitigate the impact of these parasitic infections on vulnerable populations worldwide.

### Morphology and anatomy

**Fig. 2:**
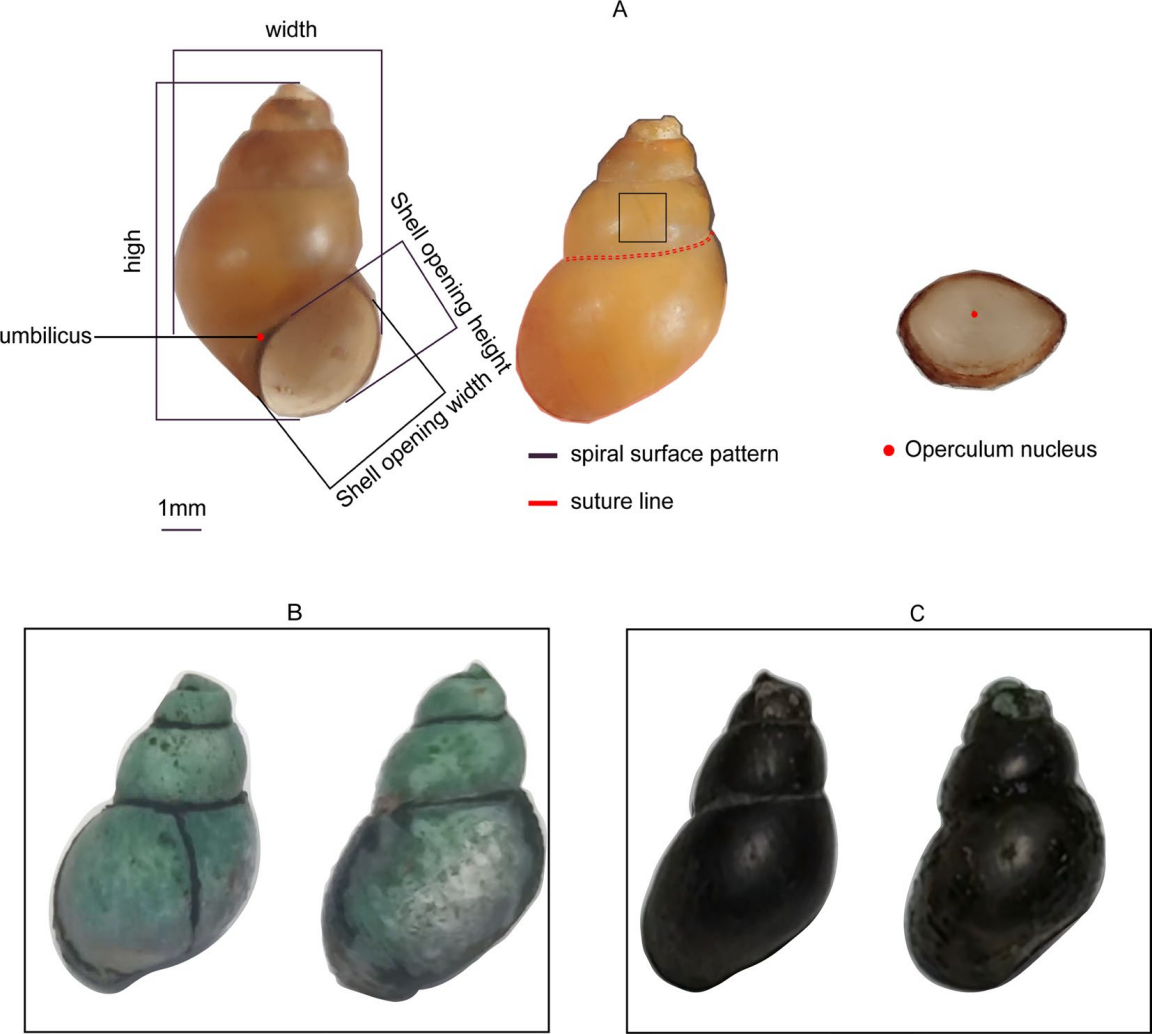
Appearance and morphology of the snail *Bithynia fuchsiana*. **A** The small red dot marks the umbilicus. The black box encloses the surface pattern of the conch shell. The broken red line segment traces the screw suture line. The large red dot signifies the nucleus of the operculum. **B** *Bithynia fuchsiana* snail growing in lotus ponds. **C** *Bithynia fuchsiana* snail growing in a ditch

The identification of snails of genus *Bithynia* traditionally relies on the examination of its external morphology and internal structure. The snail’s external morphology is mainly characterized by its shell and operculum (Fig. 2). The shell's distinctive spiral shape arises from the rotation and coiling that occurs during the snail’s development. The shell is formed by the secretion of epidermal cells of the outer membrane and comprises two distinct parts: the spire and the body whorl. The spire, composed of multiple layers, features a topmost layer known as the apex, which represents the initial embryonic shell of the organism. As the snail matures, the apex may erode, and adjacent to it lies the primary shell layer. Each layer of the snail’s shell is joined by a suture, marking the boundary between them [4]. During growth, the layers rotate around a central axis, known as the snail axis, leaving behind the umbilicus at the body snail layer. This layer, being the outermost, serves as an exit for the gastropods, with the head and feet curled within. The head is cylindrical, adorned with one or two pairs of conical tentacles on either side, while the foot is truncated anteriorly and curved posteriorly. The perimeter of the shell opening is referred to as the outer edge, whereas the region closer to the snail axis is referred to as the inner lip or inner edge. The snail secretes keratin or calcareous flakes to cover the shell opening, thereby providing protection against external threats. The surface of the shell displays various patterns, including growth lines, spirals, longitudinal ribs, keels, spines and bands. The ratio of the snail's shell layers can vary significantly due to differences in growth rates. Typically, the operculum, another defining characteristic, contains an operculum nucleus surrounded by growth lines, whose shapes vary depending on the species. When comparing different *Bithynia* species, morphological differences can be observed, as shown in Table 1. *Bithynia siamensis* has a mean (± standard deviation [SD]) shell height of 10.0 ± 1.11 mm and a mean shell width of 6.3 ± 0.60 mm, in contrast to *Bithynia misella*, which has a smaller shell with a mean height of 5.2 ± 0.59 mm and a mean width of 3.4 ± 0.34 mm. These measurements, along with presence or absence of the umbilicus and the number of spirals, serve as key distinguishing features between species. The selection of these distinguishing features or identification criteria helps to accurately differentiate between* Bithynia* species [26].

**Table 1:** Comparison of morphology and reproductive organs of different* Bithynia* species

Species name [reference]	External morphology								Internal organization	
	Shell (mm)		Aperture (mm)		Operculum (mm)		Number of whorls	Umbilicus	Reproductive system (μm)	
	Length	Width	Length	Width	Length	Width			Penis	Flagellum
*Bithynia siamensis* [26, 29]	10.0 ± 1.11	6.3 ± 0.60	4.9 ± 0.47	3.8 ± 0.35	4.24 ± 0.361	2.83 ± 0.250	5	Yes; the umbilicus is narrow and slender	4220 ± 179.5	1302 ± 106.3
*Bithynia misella* [26]	5.2 ± 0.59	3.4 ± 0.34	2.4 ± 0.21	2.1 ± 0.22	2.48 ± 0.256	1.71 ± 0.174	6	Yes; the umbilicus is prominent	1919 ± 116.6	610 ± 68.7
*Bithynia leachii* [26]	4.6 ± 0.96	3.3 ± 0.62	2.1 ± 0.38	2.0 ± 0.32	2.18 ± 0.313	1.74 ± 0.266	5	Yes; the umbilicus is deep, resembling a seam	979 ± 78.4	541 ± 48.3
*Bithynia fuchsiana* [28]	10.18 ± 0.49	5.58 ± 0.23	–	3.64 ± 0.22	–	–	5	No	3 252 ± 172.8	–

Measurements in table are presented as the mean ± standard deviation

**Fig. 3:**
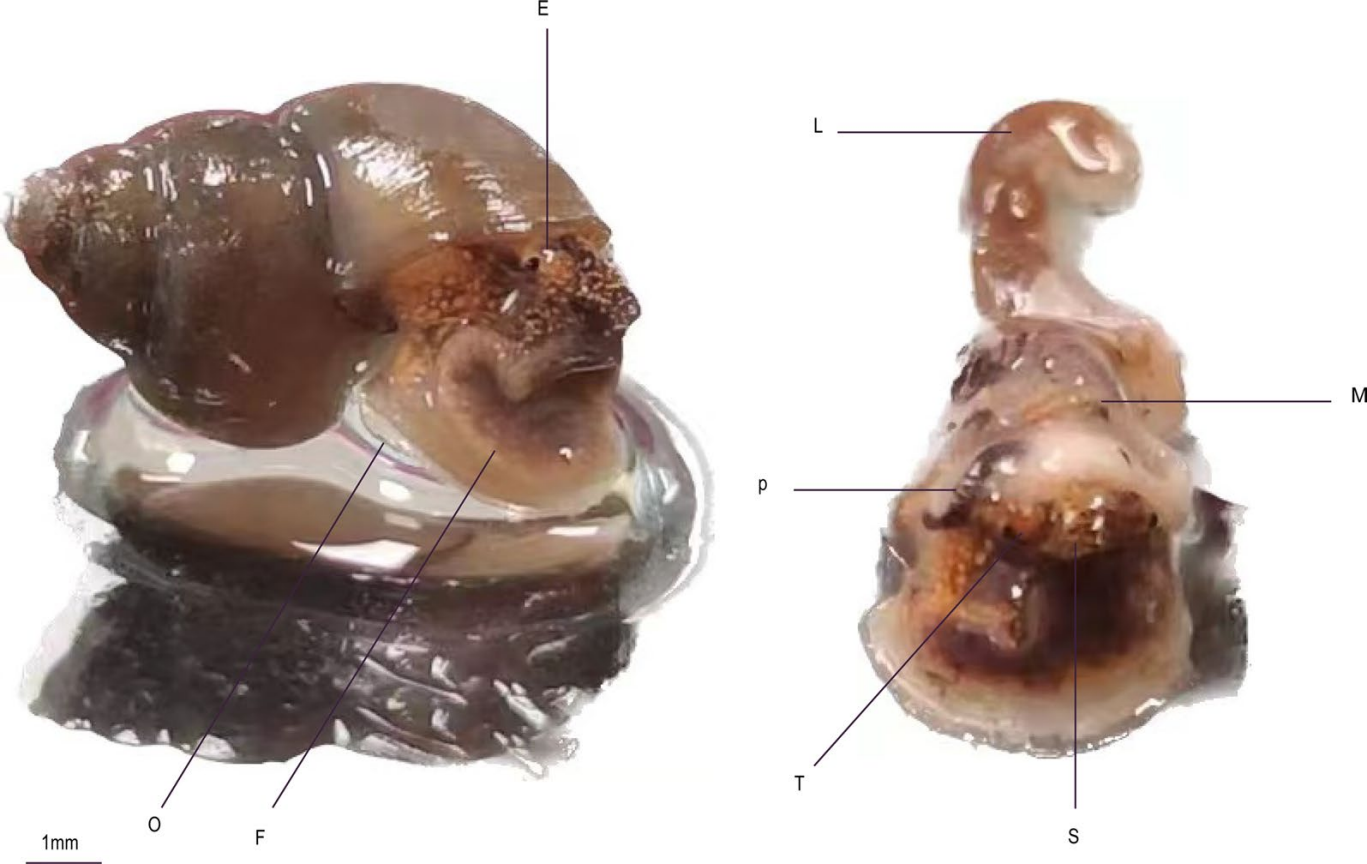
Internal organization of* Bithynia fuchsiana*. E, Eyes; F, foot; L, liver; M, mantle; O, operculum; P, penis; S, snout; T, tentacle

The internal organization of the snail, in addition to the external morphology, plays a significant role in identifying the species (Fig. 3). Snails possess six major organ systems: nervous, digestive, respiratory, circulatory, excretory and reproductive systems. Among these, radulas, which are important feeding organs in the digestive system, and reproductive organs are commonly utilized for identification purposes, with most studies focusing on these features. For example, Kim compared external morphology and internal structure within the genus *Bithynia*, reporting that only the reproductive organs of male snails exhibited interspecies differences. A subsequent investigation into radula variations revealed disparities in the number of radula morphs among species. Anatomically, all male snails belonging to the family Bithyniidae possess divided reproductive organs, but these organs do not vary within the genus *Bithynia*. The penis of *Bithynia* species is located slightly posterior to the right antennae, displaying notable differences in size and shape, both in the penis itself and its appendages. The penis is bifurcated, with a cylindrical flagellum located at its distal end [26–29]. Overall, the examination of internal structures, particularly radulas and reproductive organs, provides valuable insights for snail species identification.

In this section we focus on four species of *Bithynia* snails: *B. siamensis*, *B. misella*, *B. leachi* and *B. fuchsiana*. Data on meaningful structures for species identification in the literature were collected and shown in Table 1.

The advancements in computer science and continuous advancements in AI have greatly improved the accuracy, speed and humanitarian aspects of morphology identification. Deep learning techniques have proved to be effective in accurately identifying macroinvertebrate samples through image recognition, achieving a classification accuracy of > 95% even with a small training dataset of around 10 samples. This highlights the immense potential of image-based methods and deep learning in species identification research [30]. In recent years, programmers have developed related projects aimed at preliminary classification of certain snail species using simple methods. For example, Shi Liang successfully utilized deep learning technology to establish a visual intelligent identification model for *Oncomelania hupensis* [31, 32]. The results showed a high level of consistency between the model's identifications and those made by experts, serving as a gold standard. Nevertheless, it is important to note that the accuracy and correctness of the classification still require substantial training to be improved. Therefore, further efforts are needed to enhance the training process and ensure the validity of the classification results [31, 32].

During field operations aimed at species collection, the use of morphological methods is currently the most direct and convenient approach for species identification, typically enabling identification up to the genus level. Nonetheless, the specific molecular biology techniques for species identification may be impacted by environmental factors that can cause variations in conch shell's morphology. Firstly, environmental factors such as temperature, water quality and food availability may affect the morphological characteristics of snails, leading to variation in shell size and shape among individuals of the same species [33]. In addition, there may be an overlap of morphological features between species, making it difficult to distinguish between them on the basis of visual features alone. For example, snails of different species may show similarities in shell shape due to convergent evolution, making identification more difficult [34]. Secondly, morphological identification usually requires specialized knowledge and experience, and small differences may require tools such as microscopes for accurate observation. This limits the possibilities for non-specialists to participate in species identification and may lead to subjective results. In addition, the process of collecting and analyzing morphological characters can be time-consuming and labor-intensive, which may be impractical when a large number of samples need to be identified quickly. Furthermore, for rare or endangered species, it may not be feasible to collect a sufficient number of samples for morphological analysis.

The future of AI in morphological research is likely to see an increased focus on automating the analysis of morphological features, thereby reducing the complexity of manual operations and significantly improving work efficiency. The ability of AI to process large datasets with high precision and repeatability offers a significant advantage over traditional manual methods. Moreover, by combining machine learning and image processing technologies, AI can reveal deeper connections between morphological characteristics and genetic, ecological and evolutionary relationships [35].

The application of AI in morphological examination is not limited to basic research; it also extends to clinical diagnostics and environmental monitoring. For example, by analyzing the distribution of snails, AI can help monitor the spread of specific parasitic diseases. As AI technology continues to evolve, it is expected to play an increasingly vital role in species identification and ecological research, providing a robust foundation for the development of effective strategies to combat diseases transmitted by organisms like *Bithynia*.

In summary, the fusion of AI with morphological examination is poised to transform the way we approach species identification and ecological studies, offering a more accurate, efficient and comprehensive understanding of the natural world. The ongoing advancements in AI present a promising future for the field of morphology, with the potential to revolutionize our approach to environmental conservation and disease management.

## Multi-site enzyme electrophoresis

Multi-site enzyme electrophoresis (MEE), which is a technique that utilizes an electric field to induce the migration of enzymes in a medium, offers superior species differentiation, typing accuracy and repeatability, and is highly consistent with phylogenetic trees constructed using subsequent gene sequencing [36]. This technique primarily employs housekeeping enzymes, such as malate dehydrogenase, glucose 6-phosphate dehydrogenase, glutamate dehydrogenase and phosphoglucose translocase, which are less affected by environmental selective pressures and evolutionary convergence [37–39]. Enzyme activity can be visualized as bands on electrophoresis gels through specific histochemical staining, as each enzyme catalyzes a distinct biochemical reaction. This allows for the differentiation of species and the identification of individuals within morphologically similar or closely related populations. MEE is a valuable tool in epidemiological studies and species evolution analysis. Its applicability extends beyond traditional phenotyping methods, providing more comprehensive insights into genetic variations and relationships among organisms. It has been widely utilized in various organisms, including mammals, parasites, bacteria, fungi, among others [40–43].

The first application of MEE in identifying members of the genus *Bithynia* was by Viyanant and colleagues, who used four specific isozymes in 1985 to genotype *Bithynia* spp. and subspecies [44]. In a subsequent study in which the number of specific isozymes was increased, Saijuntha et al. found a large genetic divergence in the population* Bithynia siamensis goniomphalos* (*B.s. goniomphalos*) and a correlation between the genetic phenotypes of the parasites, suggesting the possibility of parasite-host co-evolution [45]. In turn, Kiatsopit et al. found that the large genetic differences between* Bithynia siamensis siamensis* (*B.s. siamensis*) and *B.s. goniomphalos* likely represent different species rather than currently defined subspecies [46]. These studies have led to further knowledge of the *Bithynia* genus.

MEE offers several advantages, such as its cost-effectiveness and accessibility to laboratories with limited financial resources. It allows for experimental studies to be conducted in batches, resulting in increased efficiency. The results obtained through MEE are highly reproducible and reliable, and can be applied to different species, making it a versatile technique. However, MEE does have a number of limitations. The experimental procedures involved can be cumbersome and require technicians with high technical skills. Additionally, strict conditions need to be followed for sample preparation and preservation, adding to the complexity of the technique [47].

 MEE has been widely utilized as a tool for studying genetic diversity and population structure of organisms, and it is considered to be the gold standard in population genetics studies, where allelic variation in several housekeeping genes was analyzed. This approach has contributed to the estimation of overall genotypic diversity of the species. Understanding the genetic differences in pathogen-vectored organisms through MEE is of great significance in fields such as public health, disease prevention and diagnosis and therapy [37]. Furthermore, while MEE is more accurate than morphology in terms of species identification and can provide a more in-depth analysis of population differences from a molecular biology perspective, having a basic understanding of the morphology of the snail species is essential prior to conducting MEE. Therefore, a combination of both techniques can lead to more accurate and comprehensive results.

## DNA barcoding technology

The concept of DNA barcoding was initially introduced in a publication by Arnot in 1993, with the aim to provide a species identification method based on analysis of unique DNA segments [48, 49]. A crucial primary factor in this process is the identification of a gene fragment that is universally utilized in most organisms.

The mitochondrial DNA (mtDNA) indeed offers unique advantages for genetic identification due to its strict maternal inheritance and limited recombination. This results in a more direct representation of the maternal lineage, reducing the potential for heterozygosity and sequencing errors. The cytochrome* c* oxidase subunit I (COI) gene, a popular mtDNA marker, is widely used in animal identification studies because of its significant sequence variations among different species and high evolutionary rate. These features make COI an effective tool for analyzing and identifying relationships between closely related species, subspecies and geographically distinct populations [50]. The recognition of COI as a standard gene fragment for animal DNA barcoding by the Barcode Council (now GS1 International) in 2007 underscores its importance in biodiversity studies. However, it is worth noting that the standard barcodes for plants, fungi, algae and other organisms differ from those of animals. For example, plants typically use the rbcL and matK regions as their gene barcodes, while fungi utilize internal transcribed spacer (ITS) genes. Algae, on the other hand, may have different barcodes depending on their colors [51, 52].

The application of DNA barcoding, particularly the use of COI genes, in the identification of species within the genus *Bithynia* has been a significant advancement in taxonomy. Kulsantiwong et al.’s 2013 study in Thailand highlighted the effectiveness of COI genes in distinguishing species within this genus, demonstrating its utility in resolving taxonomic ambiguities [53]. Similarly, Jiang Ying’s work in Hengzhou City, Guangxi Province, revealed that morphological similarity alone is not sufficient for species identification. The combination of morphological and genetic data, as shown in the construction of phylogenetic tree has greatly improved the accuracy of species identification [28]. Tantrawatpan et al.’s division of *B.s. goniomphalos* into three lineages provides further insights into the genetic diversity within this species [54]. Overall, the application of COI as a genetic barcoding tool for species identification complements traditional morphological classification, providing a more comprehensive and accurate understanding of biodiversity. This integration of genetic and morphological data is essential for advancing our knowledge of species boundaries and evolutionary relationships within taxonomic groups.

DNA barcoding is easy to use, quick to identify and does not demand a high level of skill by laboratory operators. Morphological characteristics provide an intuitive basis for species identification, while DNA barcoding offers genetic information at the molecular level [55]. This complementarity allows researchers to make more accurate identifications between species that are morphologically similar or difficult to distinguish by appearance. Testing can occur in large quantities, and individual genes do not change depending on the developmental stage of the individual, thereby avoiding subjective influence from researchers. Unstrict sample requirements mean that the size of the sample required is small. The method is conducive to the discovery of new species and enables a more comprehensive and reliable identification of species [56–58]. Global sharing of data lays the foundation for realizing the construction of the Earth's evolutionary tree. Despite all of these advantages, the disadvantages of DNA barcoding should not be overlooked [49]. The accuracy of DNA barcoding depends on the completeness of the reference database. For species in the* Bithynia* genus that are not yet recorded in the database, or those with incomplete records, DNA barcoding technology may not provide accurate identification results. The matrilineal inheritance of mitochondrial genes is not conducive to the identification of hybridized species. Also, the small genetic variation of new species makes the correct identification of each one questionable. Furthermore, there is a huge discrepancy between the number of genes measured so far and the number of species that exist in nature.

DNA barcoding has emerged as a powerful tool in biodiversity studies, enabling rapid and accurate species identification. By leveraging the unique properties of mtDNA and specific gene markers, DNA barcoding has the potential to revolutionize our understanding of biological diversity and ecological relationships. Nowadays, the application of DNA barcoding is not limited to species identification and classification, but has also been effectively utilized in fields such as biosafety, public health, biodiversity assessment, environmental protection, disease treatment and preservation of genetic data. Improvement in sequencing technology and the reduction in the cost of second-generation sequencing technology have made the acquisition of genetic data more convenient, which, to a certain extent, has promoted the application of DNA barcode technology [59–61].

## Metagenomics (environmental DNA)

Metagenomics, initially introduced by Jo Handelsman and colleagues, revolutionizes our comprehension of environmental genomics. Handelsman et al. contended that it is feasible to delve into the genomes of all organisms within their natural habits, and that this approach is akin to studying individual genomes [62]. Prior to this, our knowledge of individual microbial colonies was extensive, yet our comprehension of entire microbial communities remained limited [63]. Metagenomics offers us an invaluable tool to unravel the architecture of these vast microbial communities, thereby bridging this knowledge gap.

The primary applications of environmental DNA (eDNA) in the field of medically important mollusks have been centered on tracking the invasion of species within a given region and exploring the species diversity present there. However, it remains to be seen whether eDNA can be employed for the identification of species belonging to the genus *Bithynia* or for analyzing their genetic status. Despite this lack of information on *Bithynia*, numerous other invertebrate studies, along with their corresponding genetic investigations, have been successfully conducted. For example, DeMone et al. used eDNA to detect the presence of parasitic infections in shellfish [64]; ecologically diverse, species-specific eDNA testing of the Yangtze River was performed after years of a fishing ban [65]; and the spread of invasive non-native species was detected by eDNA analysis of invertebrate DNA collected at the marina by Amaral et al. [66]. Many other authors have also used eDNA in various studies [67–77].

DNA barcoding technology, akin to DNA barcoding, involves sequencing the genome of a sample for accurate identification. This approach is not only swift and efficient in determining the abundance of species but also offers numerous advantages for species identification [78]. Traditional field sampling often requires a lot of manpower and time, and it may disturb the organisms and their ecological environment. In contrast, eDNA technology, by analyzing trace amounts of DNA in environmental samples such as water and soil, can detect and quantify the presence of various organisms. This method does not require direct contact or capture of individual organisms, thereby reducing disturbance to the organisms and damage to the environment. eDNA is renowned for its speed, convenience, low cost and non-invasiveness, making it a valuable alternative or complementary method to traditional field sampling methods used in laboratory identification. Furthermore, it can be seamlessly integrated into both short- and long-term biomonitoring programs, biodiversity assessments and conservation efforts [79]. 

eDNA barcodes, however, although valuable, are not without their limitations [80]. Firstly, the mere presence of a species’ DNA in the environment does not unequivocally indicate the existence of that species, as it could have been introduced by predators such birds, whose droppings may carry DNA from species in other regions [81]. Secondly, DNA detected in soil may belong to species that existed long ago but whose DNA has persisted due to degradation in the environment, resulting in lower retention rates, especially in warmer regions [71]. The movement of DNA in water with the current complicates the accurate determination of the geographic location of a species community [82]. Furthermore, eDNA does not prioritize the analysis of intraspecific genetic variation, limiting the detection and analysis haplotypes to the mitochondrial genomic region [83, 84]. Therefore, the entire process requires standardization, including uniform steps for sample collection from diverse environments, optimization of collection and extraction methods, guaranteed sample purity [85], primer design [86] and the development of comprehensive reference databases. Each step in data processing and results interpretation can significantly impact conservation decisions, highlighting the need for utmost care and precision in the entire process [87].

Until now, the most extensively explored applications of eDNA have centered around ecosystem and biodiversity monitoring. This involves the meticulous analysis of individual species within the environment, with the analysis tailored to the specific population of species being studied [78]. For comprehensive ecosystem studies, eDNA enables the detection and analysis of all species throughout the environment [81], with their DNA taxonomically identified through high-throughput sequencing. In essence, this technology allows for the detailed analysis of both individual populations and entire communities. Currently, global warming and anthropogenic factors are having a profound impact on the entire ecosystem, posing a significant threat to the diversity of species on Earth. Species are becoming extinct at an alarming and unprecedented rate. In this context, eDNA barcoding technology stands out as a potent tool for assessing species diversity, providing crucial insights into the health and sustainability of our natural world.

In recent years, there has been a plan in Guangxi, an autonomous region in southern China, to build a canal, the Pinglu Canal, to open up the Yujiang and Qinjiang river systems; this region is a high prevalence area for clonorchiosis. The opening of the Pinglu Canal may lead to the proliferation of clonorchiosis (from the endemic system of the Yujiang River to the non-endemic system of the Qinjiang River) [88] and to the extension of the habitat range of the snail. The use of eDNA to test the water samples from the canal can be used to provide advance warning for the monitoring of the disease's proliferation. However, the implementation of such a monitoring program faces may challenges. Firstly, when studying the role of *Bithynia* species in the transmission of liver fluke diseases, it is necessary to continuously monitor the dynamics of disease transmission, which may require the combination of traditional epidemiological surveys and eDNA technology; secondly, human activities, such as the construction of water conservation systems, may alter the distribution of *Bithynia* species and the transmission patterns of liver fluke diseases [89]. For example, the planned construction of the Pinglu Canal in Guangxi may affect the spread of the disease, but the successful application of eDNA technology depends on a comprehensive reference database, and for some species of *Bithynia*, there may not be detailed records in the database, which limits the application of eDNA technology in species identification, among other challenges.

To achieve more comprehensive species identification and ecological monitoring, eDNA, DNA barcoding and morphological analysis can be employed in tandem [90]. eDNA technology, which analyzes environmental DNA from water samples, can detect and identify the presence of specific species, offering a particular advantage for monitoring those that are challenging to capture or observe. DNA barcoding technology utilizes short, variable DNA fragments from within organisms that are standardized and readily amplifiable, enabling rapid and accurate species identification. Morphological analysis classifies species by observing and comparing their physical characteristics. Combining these three methods can improve the accuracy and efficiency of species identification, while providing richer data for ecological monitoring. This integrated approach not only improves our understanding of the diversity and distribution of *Bithynia* species but also assesses and predicts the impact of human activities on ecosystems, thereby providing a scientific basis for biodiversity conservation and disease management.

## Outstanding questions

Among the three hosts of *C. sinensis*, the first intermediate host, snails, has garnered surprisingly little attention. Current understanding of the incubation and development of liver fluke eggs within snails remains hazy, with some studies indicating a peak infection rate at 28 °C, although this finding lacked statistical significance when compared to temperatures of 24 °C and 26 °C. Once ingested at an optimal temperature, the eggs hatch into miracidia within the snail’s intestine, subsequently developing into sporocysts and rediaes in 30 days. The development of cercariae, however, is a more protracted process, taking over 90 days to emerge from the snail’s tail and proceed to infect the next host. Notably, the release of cercariae is episodic and ceases when the temperature dips to below 20 °C [91, 92]. The number of infected eggs has been found to correlate closely with both the infection rate and the survival rate of snails. Intriguingly, studies have shown no discernible difference in infection rate between snails with a snail-to-egg ratio of 1:60 and those with a ratio of 1:90. Furthermore, the potential link between the snail’s sex and susceptibility to *C. sinensis* infection remains a mystery [93]. While it is widely acknowledged that numerous members of this genus can serve as hosts for liver flukes, the taxonomic identification of these species themselves is shrouded in uncertainty. Significant ambiguity remains regarding the survival strategies, reproductive patterns, migratory habits, origin and ecological positioning of these snails. Accurate taxonomic classification is paramount to enabling comprehensive and thorough investigations in this domain. All of the above challenges demonstrate that current knowledge of the genus* Bithynia* is still very much inadequate, and some of the methods described in the preceding sections are the key to opening the door to this unknown world.

However, integrating morphological and molecular data presents its own set of challenges. For example, discrepancies may arise due to variations in environmental conditions that affect morphological traits, or due to genetic mutations that do not correspond to visible characteristics. Additionally, the quality of molecular data can be compromised by factors such as DNA degradation or contamination, which can then lead to erroneous conclusions. Furthermore, the interpretation of molecular data requires sophisticated bioinformatics tools and expertise, which may not be readily available in all research settings. Despite these challenges, the integration of both morphological and molecular approaches is crucial for a holistic understanding of species diversity and evolution within the* Bithynia* genus.

**Fig. 4:**
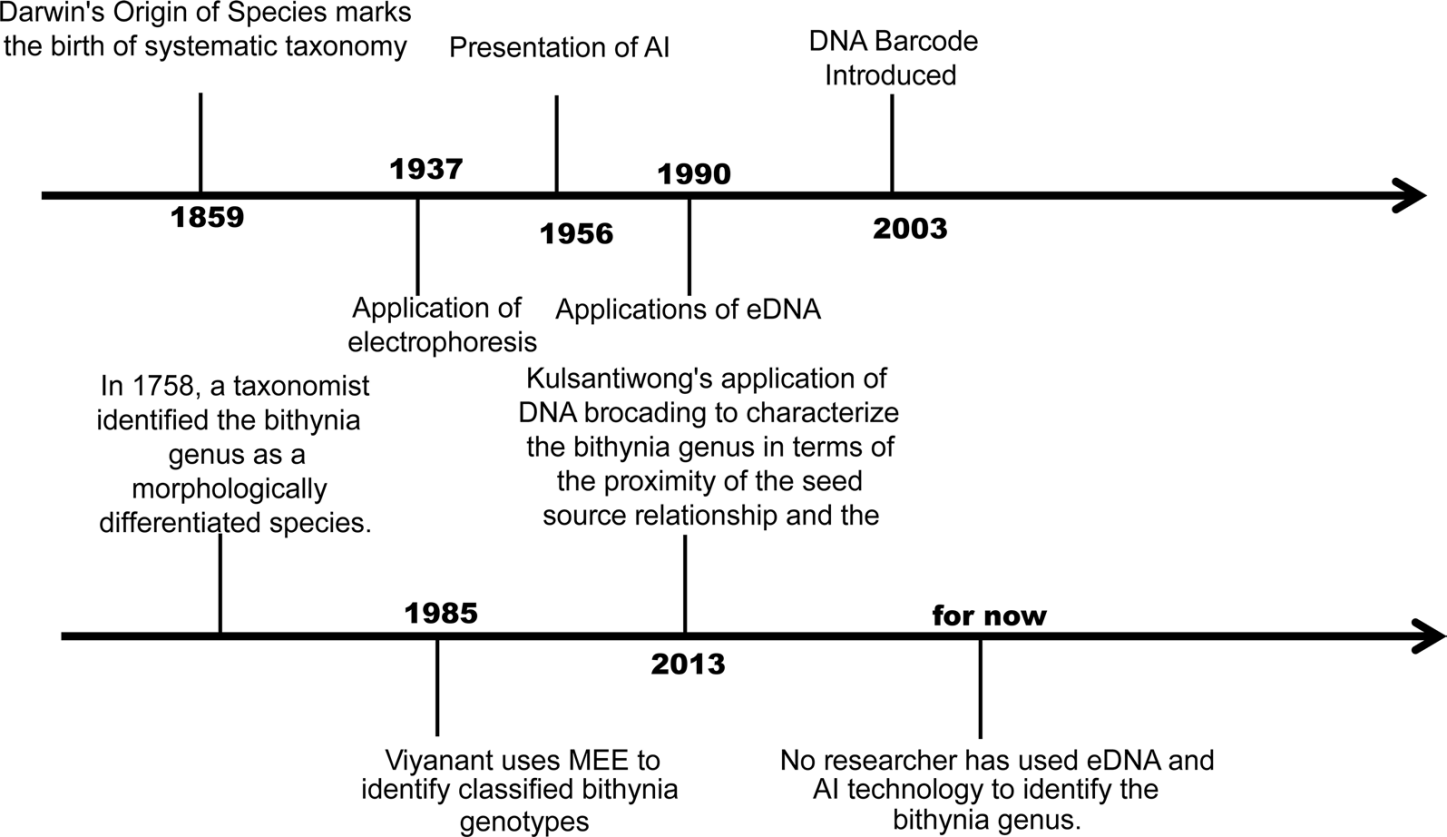
Timeline of the emergence and application of new technologies. The accompanying text at each time point delves into the precise timing of the emergence of the technologies and their application regarding the *Bithynia* genus. AI, Artificial intelligence; eDNA, environmental DNA; MEE, multi-site enzyme electrophoresis

Technological advancements have significantly improved the taxonomic identification of species, incorporating both morphological features and cellular molecules. Each species exhibits distinct characteristics that are integral to its taxonomic classification. It is precisely these morphological and molecular differences among species that need to be studied and observed. During the non-molecular era, the classification and identification of *Bithynia* genus primarily relied on morphological appearance, analysis of the reproductive system and the counting of radulae [94]. However, with the vast number of species on earth numbering in the tens of thousands, the sole reliance on human observation of morphology significantly diminishes the accuracy of species identification and often leads to misjudgment [95, 96]. Fortunately, the advent of molecular biology technology has revolutionized this process, enabling the accurate comparison and classification of species at the molecular level. Initial molecular techniques focused on protein-level exploration (MEE), which laid the foundation for and was quickly followed by genetic-level analyses, including eDNA and DNA barcoding. These advancements have not only deepened our understanding of species relationships but have also significantly strengthened the field of molecular taxonomy [96–98] (Fig. 4). By examining the evolution of life from a molecular perspective, we can advance the cognitive classification and identification of species worldwide, unhindered by morphological limitations. Each of the molecular techniques mentioned herein, namely morphology, MEE, DNA barcoding and eDNA, possesses its own unique strengths and challenges, and is tailored to specific applications. Morphology offers cost-effective species identification, while MEE and DNA barcoding provide genotype differentiation and enriched species data, albeit accompanied by higher financial and greater technical requirements [99]. eDNA stands as a non-invasive method for biodiversity data collection, and it necessitates expertise in environmental science and molecular biology. Each technique serves a distinct purpose: morphology is ideal for field and laboratory-based studies; MEE excels for genotype differentiation; DNA barcoding captures genetic data; and eDNA facilitates rapid and comprehensive collection of biodiversity data [81]. However, all of these methods require skilled personnel and are susceptible to limitations stemming from human factors, technical proficiency and control over experimental conditions. The choice of the appropriate technique depends on a range of considerations, including research objectives, availability of resources and technical expertise capabilities. Therefore, in studies on species identification, different methods are often employed on varying occasions or a combination of techniques are utilized to achieve the desired outcomes. In the present study, we have compiled a thorough list and conducted a comprehensive analysis of each method and technique, as shown in Table 2. Furthermore, we have undertaken a comparative evaluation of diverse methodologies, with the aim to provide a valuable reference for further examination.

**Table 2:** Comparison of different methods for the identification of biological samples

Factors	Methods			
	Morphology [4]	Multi-site enzyme electrophoresis [38–40, 47]	DNA barcoding [50–53]	Environmental DNA [64–67]
Time	< 10 min	3–5 h	1–2 days	1–2 days
Cost	Low	Costs more than morphology	Costs more than MEE	Costs more than DNA brocading
Sampling requirements	1. Collect a complete biological sample 2. Record detailed collection information 3. Preserve samples using appropriate fixatives	1. Keep the sample at a low temperature 2. Collect sufficient biological material 3. Avoid cross-contamination between samples	1. Collect high-quality DNA samples 2. Samples should be stored at low temperatures 3. Ensure that the sample volume is sufficient for accurate sequencing	1. Collect water or environmental samples 2. Use sterile containers and tools 3. Record precise sampling locations and conditions
Key technology and/or tool(s)	*Key techniques*: microscope observation, morphometric measurements, image analysis *Tools*: optical microscope, measuring tools (calipers), image processing software, camera *Statistical analysis methods*: ANOVA, non-parametric tests, independent samples t-tests	*Key technology*: protein extraction and purification, protein electrophoresis *Tools*: electrophoresis equipment, gel imaging system	*Key technology*: DNA extraction, PCR amplification, sequencing, sequence comparison *Tools*: thermal cycler, sequencer, DNA barcode database, bioinformatics analysis tools (e.g. OBItools package, MAGE, BLAST)	*Key technology*: environmental sample collection, DNA extraction, high-throughput sequencing, bioinformatics analysis *Tools*: sterile sampling equipment, DNA extraction reagents, high-throughput sequencing platform, bioinformatics analysis tools (EcoView, BOLD Systems)
Applications	*Implementation environment*: measurements can be observed in the field and are usually carried out in a laboratory setting, where microscopes and measuring tools are needed to observe and compare morphological characteristics of species *Target objects*: comparative morphological characters of different species within the genus* Bithynia* *Application scenarios*: commonly used in taxonomic studies at universities and research institutes	*Implementation environment*: performed in a laboratory, requiring electrophoresis equipment and associated reagents to separate and characterize proteins *Target objects*: protein differences in species of the genus Bithynia, distinguishing similar species *Application scenarios*: For Disease Control Departments and research institutions	*Implementation environment*: performed in the laboratory, it requires DNA extraction, PCR amplification and sequencing equipment, as well as bioinformatics tools for data analysis *Target objects*: DNA sequence analysis of species of the genus* Bithynia* for precise species identification *Application scenario*s: Universities and research institutes for biodiversity monitoring, ecological research and invasive species detection	*Implementation environmen*t: performed in the laboratory or in the field, it requires sterile sampling tools and high-throughput sequencing platforms, as well as bioinformatics tools for data analysis *Target objects*: detection and identification of species of the genus Bithynia from environmental samples, including rare or hard-to-catch species *Application scenarios*: suitable for biodiversity assessment in nature reserves and routine surveillance in disease control departments
Experimental requirements	No need for complex instruments	Requires basic electrophoresis and extraction equipment	Advanced DNA synthesis kit, basic laboratory equipment, high requirements for PCR and sequencing	Sampling equipment, standard laboratory tools, high-throughput sequencing platform requirements
Technical personnel requirements	Sufficient grasp of basic morphology for study object	Adequate proficiency in basic molecular biology techniques	Sufficient proficiency in molecular biology techniques and analysis software	Adequate skills in basic molecular biology and relevant analysis software
Discrimination capacity	Accurate species identification based on distinct morphological features	Accurate differentiation of genotypes and similar species in populations	Obtains genetic data from population, offering more species information and improved identification accuracy	Non-invasively obtains environmental samples, enabling rapid, comprehensive, and accurate biodiversity data collection
Limitations	*Limitations*: reliance on expertise makes it difficult to distinguish between morphologically similar species *Challenges*: environmental changes may affect species morphology	*Limitations*: requires specific equipment and high purity protein samples *Challenges*: results are susceptible to sample handling and experimental conditions	*Limitations*: reliance on high-quality DNA samples and well-established reference databases *Challenges*: technically demanding, possible PCR amplification bias, sample contamination	*Limitation*s: high requirements for sample collection and processing, susceptibility to environmental factors *Challenges*: need for robust bioinformatics tools to analyze data and prevent cross-contamination

*ANOVA* Analysis of variance,* BLAST* Basic Local Alignment Search Tool,* BOLD* Barcode of Life Data, *MEE* Multi-site enzyme electrophoresis

In summary, the genus *Bithynia* serves as a pivotal intermediary host in the transmission of liver fluke diseases, emphasizing the paramount importance of its taxonomic identification for comprehension of disease transmission mechanisms. Future research should focus on the integration of traditional morphological methods with cutting-edge molecular techniques to tackle these challenges. By harnessing the synergies of both approaches, researchers can attain a more precise and holistic taxonomic identification of members of the *Bithynia* genus, which will ultimately contribute to a deeper understanding of the ecological impacts of the host and the dynamics of associated disease transmission. The exploration of immunobiological responses in Bithynia species to parasitic infections, such as those due to infection by *O. viverrini* and *C. sinensis*, could open new avenues in comparative immunology and provide insights into host-parasite co-evolution. Additionally, the application of genomics and gene editing technologies may offer novel strategies for disease control and prevention by disrupting the life-cycle of the parasite within the snail host [100].

The development of drugs and vaccines targeted at the parasite could be accelerated by leveraging the immunological insights gained from research on *Bithynia* spp., potentially leading to new therapeutic interventions [5]. Incorporating AI and machine learning into the analytical pipeline may enhance the efficiency and accuracy of species identification, facilitating real-time monitoring and management of disease outbreaks. Furthermore, the discovery and characterization of new* Bithynia* species in understudied regions could broaden the taxonomic landscape, providing fresh insights into the biodiversity and evolutionary history of these ecologically significant snails [101].

These concerted efforts will not only bolster the fundamental science underpinning research on* Bithynia* but also have practical implications for public health and environmental conservation programs. The future of *Bithynia* research is poised to be multidisciplinary, integrating advances in molecular biology, immunology, genomics and computational biology to unravel the complexities of host-parasite interactions and to inform strategies for the mitigation of liver fluke diseases.

## Conclusions

In conclusion, snails in the genus *Bithynia*, as intermediate hosts for *C. sinensis*, are crucial for the life-cycle of the liver fluke and the transmission of associated diseases. Despite the challenges in taxonomic classification and the integration of morphological and molecular data, accurate identification of *Bithynia* species is essential for understanding the epidemiology of these parasites. Future research should continue to employ a combination of traditional and modern molecular techniques to enhance current understanding of the genus's diversity and its role in disease transmission. Exploration of the immunological responses of *Bithynia* spp. to parasitic infections could reveal new aspects of host-parasite co-evolution and inform strategies for disease control. Additionally, the integration of AI and machine learning in taxonomic studies may improve the precision and efficiency of species identification, with implications for public health and conservation efforts. Study of the genus *Bithynia* is set to be a multidisciplinary endeavor, harnessing insights from molecular biology, immunology, genomics and computational biology to address the complexities of host-parasite interactions and to develop effective mitigation strategies against liver fluke diseases.

## Data Availability

No datasets were generated or analyzed during the current study.

## Abbreviations

AI: Artificial intelligence
*B.s. goniomphalos*: *Bithynia siamensis goniomphalos*
*B.s. siamensis*: *Bithynia siamensis siamensis*
eDNA: Environment DNA
MEE: Multi-site enzyme electrophoresis
